# Efficacy and safety of stem cell therapy in patients with dilated cardiomyopathy: a systematic appraisal and meta-analysis

**DOI:** 10.1186/s12967-019-1966-4

**Published:** 2019-07-11

**Authors:** Shu-Ling Rong, Ze-Kun Wang, Xue-Dong Zhou, Xiao-Lin Wang, Zhi-Ming Yang, Bao Li

**Affiliations:** 1 Department of Cardiology, The Second Hospital of Shanxi Medical University, Taiyuan, Shanxi People’s Republic of China; 2 State Key Laboratory of Oral Diseases, Department of Conservative Dentistry and Endodontics, West China Hospital of Stomatology, Sichuan University, Chengdu, Sichuan People’s Republic of China; 3 Department of Neonatology, The Second Hospital of Shanxi Medical University, Taiyuan, Shanxi People’s Republic of China

**Keywords:** Stem cell therapy, Dilated cardiomyopathy, Meta-analysis, Systematic appraisal

## Abstract

**Background:**

The clinical significance of stem cell therapy in the treatment of dilated cardiomyopathy remains unclear. This systemic appraisal and meta-analysis aimed to assess the efficacy and safety of stem cell therapy in patients with dilated cardiomyopathy. After searching the PubMed, Embase, and Cochrane library databases until November 2017, we conducted a meta-analysis to evaluate the efficacy and safety of stem cell therapy in patients with dilated cardiomyopathy.

**Methods:**

The weighted mean difference (WMD), standard mean difference (SMD), relative risk (RR), and 95% confidence interval (CI) were summarized in this meta-analysis. Both fixed effects and random effects models were used to combine the data. Sensitivity analyses were conducted to evaluate the impact of an individual dataset on the pooled results.

**Results:**

A total of eight randomized controlled trials, which involved 531 participants, met the inclusion criteria in this systematic appraisal and meta-analysis. Our meta-analysis showed that stem cell therapy improves left ventricular ejection fraction (SMD = 1.09, 95% CI 0.29 to 1.90, *I*^2^ = 92%) and reduces left ventricular end-systolic volume (SMD = − 0.36, 95% CI − 0.61 to − 0.10, *I*^2^ = 20.5%) and left ventricular end-diastolic chamber size (SMD = − 0.48, 95% CI − 0.89 to − 0.07, *I*^2^ = 64.8%) in patients with dilated cardiomyopathy. However, stem cell therapy has no effect on mortality (RR = 0.72, 95% CI 0.50 to 1.02, *I*^2^ = 30.2%) and 6-min-walk test (WMD = 51.52, 95% CI − 24.52 to 127.55, *I*^2^ = 94.8%).

**Conclusions:**

This meta-analysis suggests that stem cell therapy improves left ventricular ejection fraction and reduces left ventricular end-systolic volume and left ventricular end-diastolic chamber size in patients with dilated cardiomyopathy. However, future well-designed large studies might be necessary to clarify the effect of stem cell therapy in patients with dilated cardiomyopathy.

## Background

Cardiomyopathies represent a complex group of heterogeneous heart muscle diseases caused by mechanical and/or electrical dysfunction, usually manifesting as inadequate ventricular hypertrophy or dilatation [1]. Dilated cardiomyopathy is characterized by the presence of left ventricular chamber enlargement and contractile dysfunction without abnormal stress conditions and severe coronary artery disease [2, 3]. Dilated cardiomyopathy is the third major cause of heart failure and the most frequent indication for heart transplant worldwide, with a prevalence of 40 in 100,000 persons and an annual incidence of seven in 100,000 persons [1, 4, 5]. Dilated cardiomyopathy can occur irrespective of the sex and ethnic group; in dilated cardiomyopathy, the systolic capacity of the left ventricular decreases and the patient often suddenly dies; about half of the people are reported to have died within 5 years of diagnosis [6].

Current treatments for dilated cardiomyopathy, such as beta-blockers, angiotensin receptor antagonists, angiotensin-converting enzyme inhibitors, and mechanotherapy, are aimed at reducing the rate of damage to the myocardium and not increasing its regenerating potential. Thus, the development of new therapeutic methods for this condition is required for this significant, unmet medical need. Over the last one and half decades, several stem cell studies have been conducted in patients with cardiovascular disease who underwent autologous and allogeneic stem cell transplantation using various stem cell types and have used numerous strategies for the management of stem cell deficiency [7]. Autologous bone marrow stem cell implantation in coronary artery has been proven to be safe and effective in improving the cardiac function of patients with infarction and chronic ischemia in preclinical and clinical conditions [8, 9]. Greatly reduced coronary blood flow reserve and restricted microvascular function have been found in patients with dilated cardiomyopathy [10, 11]. Several clinical studies have shown the safety of cell therapy in these patients [12, 13], although there’s still some controversy over the underlying mechanisms [14–16] and specific procedures at the methodological level [13, 17]. To provide a comprehensive assessment of the effects of stem cells therapy in patients with dilated cardiomyopathy, we aimed to perform a systematic appraisal and meta-analysis of published studies.

## Methods

The present meta-analysis was performed in accordance with the Preferred Reporting Items for Systematic Reviews and Meta-analysis guidelines [18].

### Search strategy

We searched for relevant studies in the Embase, PubMed and Cochrane Library databases from January 1990 to November 2017. The following search terms were used: “dilated cardiomyopathy”, “dilative cardiomyopathy”, “stem cell”, “bone marrow cells”, “mesenchymal stem cell”, “hematopoietic stem cells”, “progenitor stem cell”, “mother cells”, and “colony forming units”. Two reviewers independently searched the above mentioned databases, and the third reviewer resolved all disputes about eligibility. The article search was not limited by study design; however, only articles published in English were searched. All scanned summaries, studies, and quotations were reviewed. In addition, a manual cross retrieval of the bibliography of the retrieved manuscripts was conducted to further search for relevant publications.

### Selection criteria

Studies that (1) involved patients with dilated cardiomyopathy; (2) used at least two comparison groups, that is, one group that received stem cell therapy/transplantation and another group that received control treatment without stem cell therapy/transplantation; (3) were published in English; and (4) used mortality and left ventricular ejection fraction (LVEF) as primary outcome measures and left ventricular end-systolic volume (LVESV), left ventricular end-diastolic chamber size (LVEDCS), and 6-min-walk test as second outcome measures were included. Studies that (1) used the same population or overlapping database and (2) were performed in animal models were excluded.

### Data extraction and quality assessment

Two researchers independently extracted data from individual studies based on the descriptions provided by the authors of the included studies. Any disagreement was resolved by discussion, and a third author was consulted where necessary. The following data were obtained from each article: first author, year of publication, country, mean age, intervention, time of follow-up, study design, and outcomes assessed. When results were presented in a figure, we used GetData Graph Digitizer 2.25 (http://getdata-graph-digitizer.com) to determine the exact values. The quality of randomized controlled trials (RCTs) was assessed using the Cochrane Collaboration’s tool for assessing risk of bias [19]. The assessment included the following components: random sequence generation, blinding of patients and study personnel, allocation concealment, blinding of outcome assessment, selective reporting of outcomes, completeness of outcome data, and other threats to validity.

### Statistical analysis

The meta-analysis was performed using Stata 12.0 (Stata-Corp, College Station, TX, USA). The standard mean difference (SMD)/weighted mean difference (WMD) and 95% confidence intervals (CI) were calculated for the continuous data, and the risk ratio (RR) and 95% confidence intervals were calculated for dichotomous data. Q-statistics and *I*^2^ index were used to evaluate the heterogeneity between various effects. The heterogeneity was at *I*^2^ > 50%, which was statistically significant. The random-effects model was used in the analysis. By contrast, we used a fixed-effects model to calculate the summary effect. We conducted a series of sensitivity analyses to estimate the influence of each study by omitting one study at a time. We used STATA 12.0 software to perform Begg’s and Egger’s test to quantify the publication bias. Significant publication bias was defined as a two-sided *P* value of < 0.05, which was statistically significant.

## Results

### Study selection

**Fig. 1:**
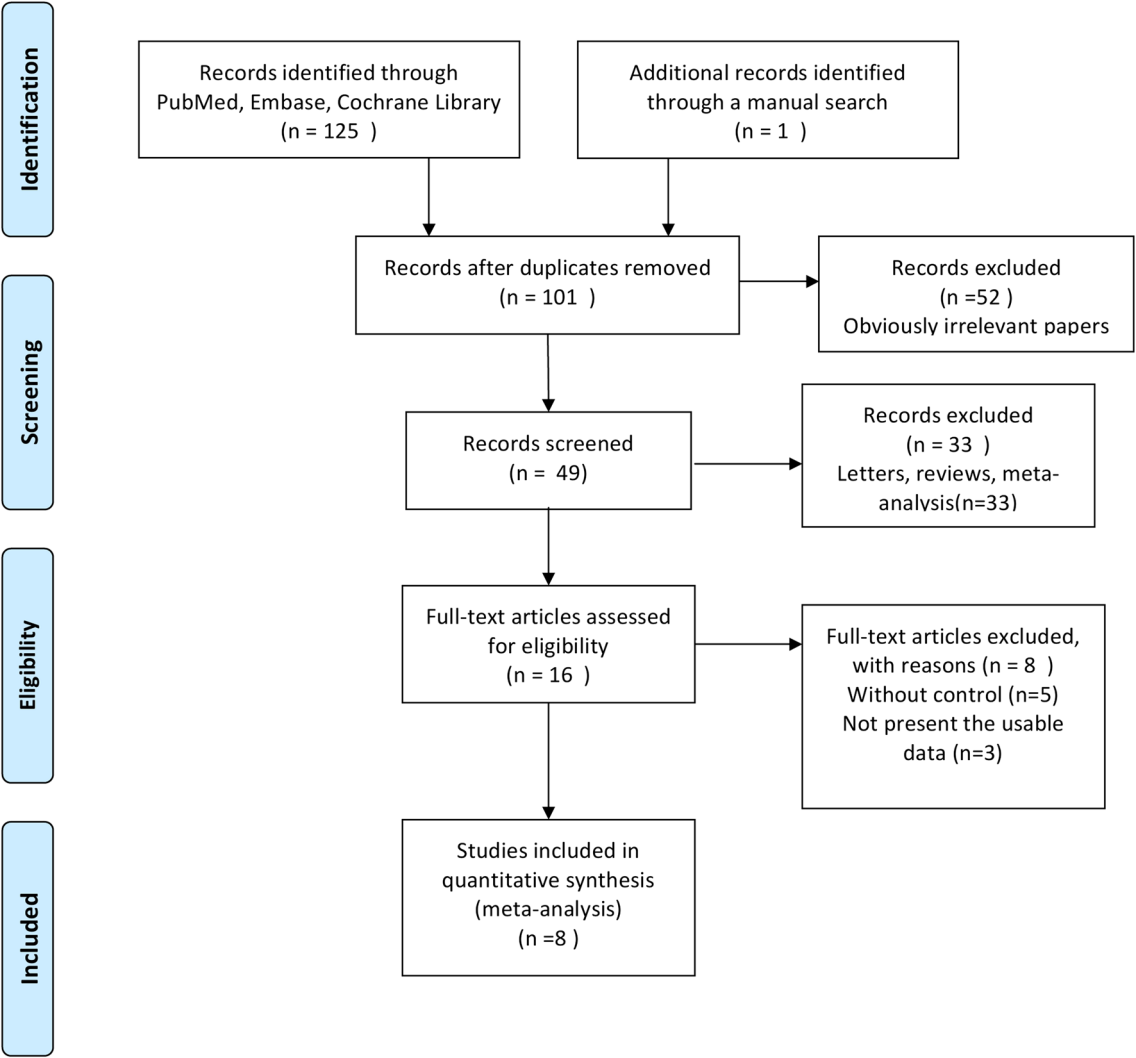
Flow diagram of studies identification

As demonstrated in Fig. 1, from our electronic search, we identified 125 studies. We found one additional study by cross-referencing the reference lists of other relevant articles. According to the inclusion criteria, 101 studies were retained after removing the duplicates. Fifty-two articles, whose titles or abstracts were screened, were excluded as the studies were irrelevant. Of the remaining 49 articles, 33 were excluded as they were categorized as letters, reviews, and meta-analyses. The remaining 16 studies were evaluated in detail. Eight of these studies were excluded, of which five had no control group and three did not present the usable data. As a result, only eight randomized controlled trials [14, 20–26] with 524 participants that fulfilled our inclusion criteria were analyzed.

### Characteristics of the studies

**Fig. 2:**
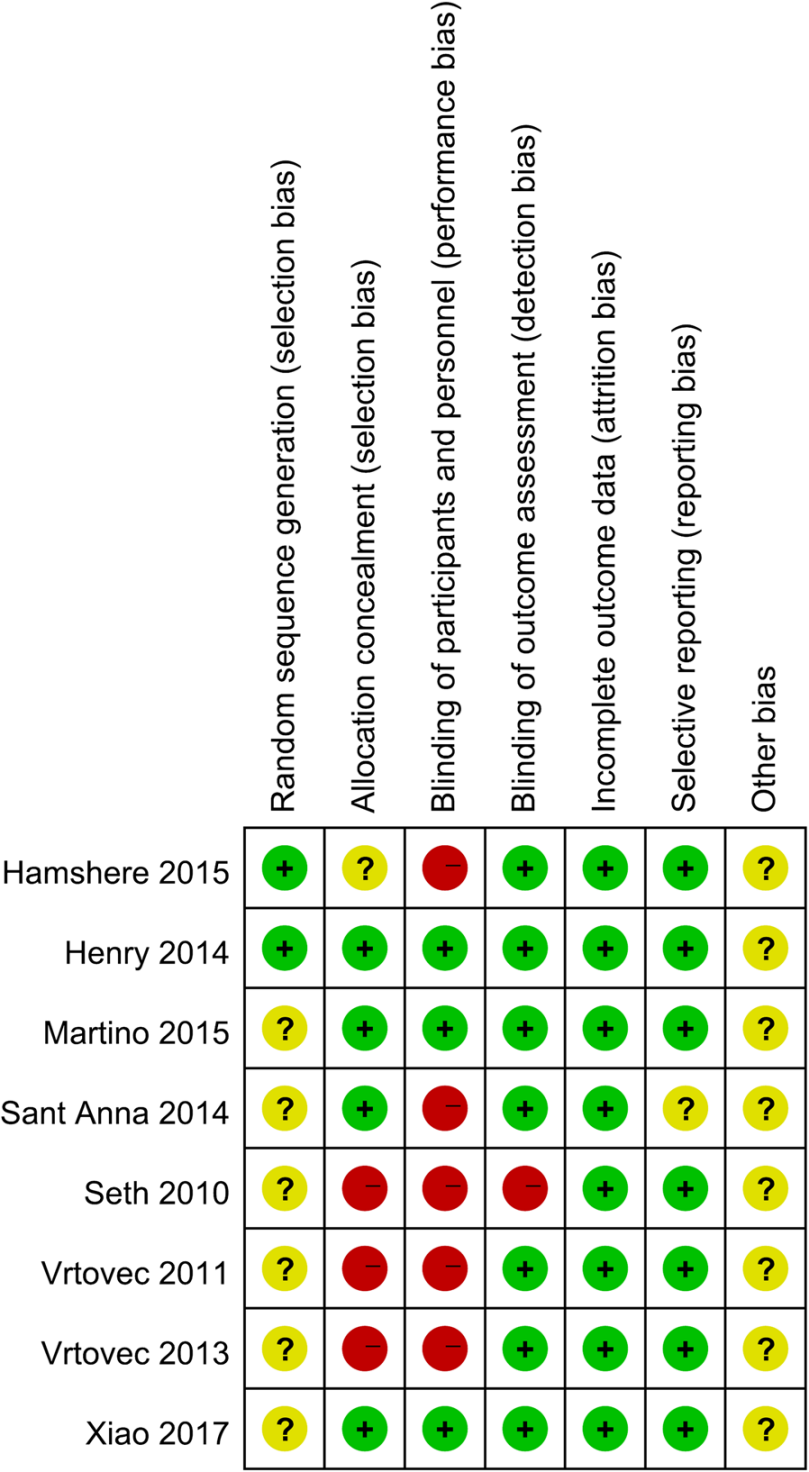
Risk of bias summary for the randomized trials included in the meta-analysis. Symbols: (+): low risk of bias; (?): unclear risk of bias; (−): high risk of bias

**Fig. 3:**
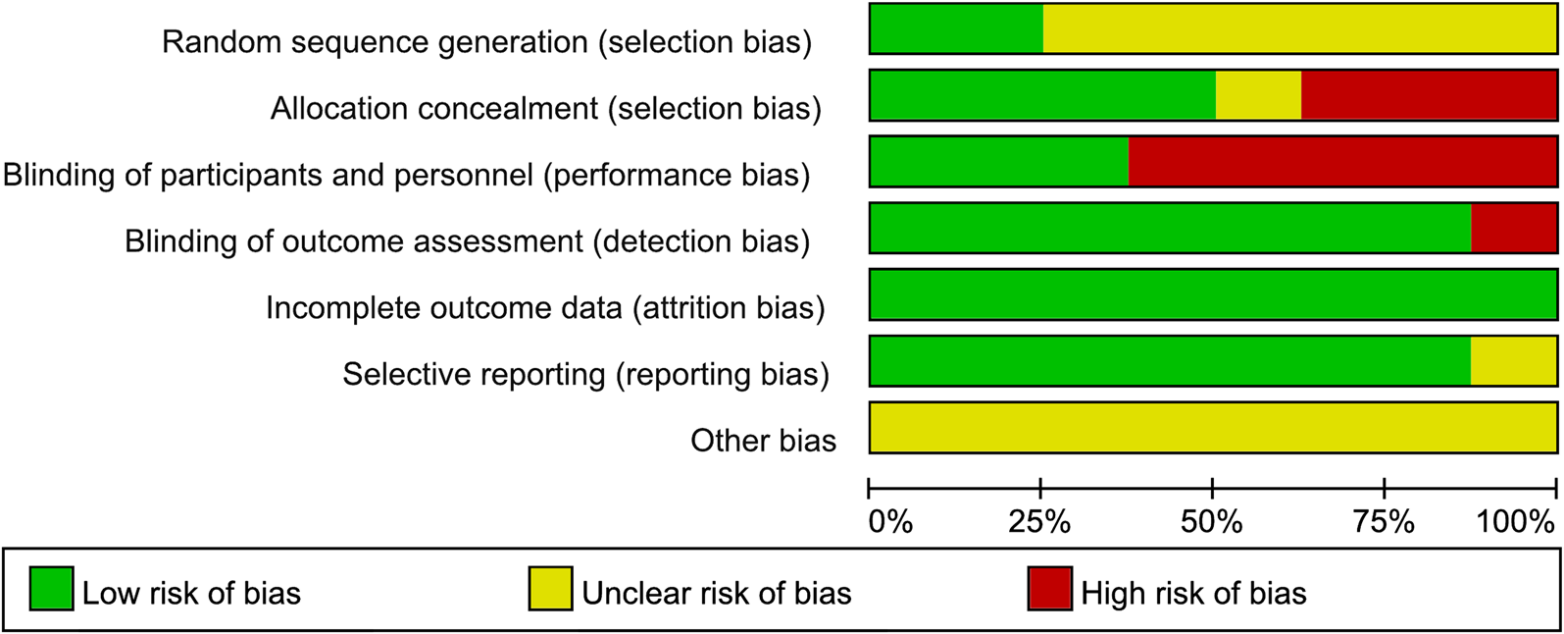
Risk of bias graph for the randomized trials included in the meta-analysis

The eight RCTs assessed 531 participants, including 276 participants who received stem cell therapy and 255 controls. The characteristics of the studies are shown in Table 1. The included articles were published between 2010 and 2017. The average age of patients in each trial ranged from 45 to 57.9 years old (Table 1). We also used a tool recommended by the Cochrane Collaboration to assess for risk of bias. A graph and summary of selection bias, detection bias, performance bias, reporting bias, attrition bias, and other bias identified in each RCT are shown in Figs. 2 and 3. Three studies lacked allocation concealment, five studies lacked blinding to participants, and one study lacked blinding to outcome assessment.

**Table 1:** Characteristics of the studies included in this meta-analysis

Authors/year of publication	Country	Male (%)	Mean age	Intervention		Follow-up	Study design	Outcomes assessed
				Stem cell	Control			
Seth/2010 [20]	India	83.9	Stem cell: 45 ± 15 years Control: 49 ± 9 years	41	40	36 M	RCT	Mortality, LVEF, LVEDCS, and LVESV
Vrtovec/2011 [14]	USA	79	53 ± 9 years	28	27	12 M	RCT	Mortality, LVEF, LVEDCS, and 6-min-walk test
Vrtovec/2013 [21]	USA	81	54 ± 9 years	55	55	60 M	RCT	Mortality, LVEF, LVEDCS, and 6-min-walk test
Henry/2014 [22]	USA	68.9	Stem cell: 57.9 ± 11 years Control: 52.3 ± 11 years	18	11	12 M	RCT	Mortality, LVEF, LVESV, and 6-min-walk test
Sant Anna/2014 [23]	Brazil	60	Stem cell: 48.3 ± 8.71 years Control: 51.6 ± 7.79 years	20	10	12 M	RCT	Mortality, LVEF, LVESV, LVEDCS, and 6-min-walk test
Hamshere/2015 [26]	UK	75.8	Stem cell: 57.67 ± 12.32 years Control: 56.79 ± 9.84 years	15	14	12 M	RCT	Mortality, LVEF, LVEDCS, and LVESV
Martino/2015 [24]	Brazil	70.6	Stem cell: 51 ± 11.1 years Control: 49.6 ± 11.1 years	82	78	12 M	RCT	Mortality, LVEF, LVESV, and 6-min-walk test
Xiao/2017 [25]	China	75.6	Stem cell: 51.6 ± 12.2 years Control: 54.4 ± 11.6 years	17	20	12 M	RCT	Mortality, LVEF, LVEDCS, and 6-min-walk test

*LVEDCS* left ventricular end-diastolic chamber size, *LVEF* left ventricular ejection fraction, *LVESV* left ventricular end-systolic volume, *M* months, *NA* not available, *RCT* randomized controlled trial

### Quantitative synthesis

#### Mortality

**Fig. 4:**
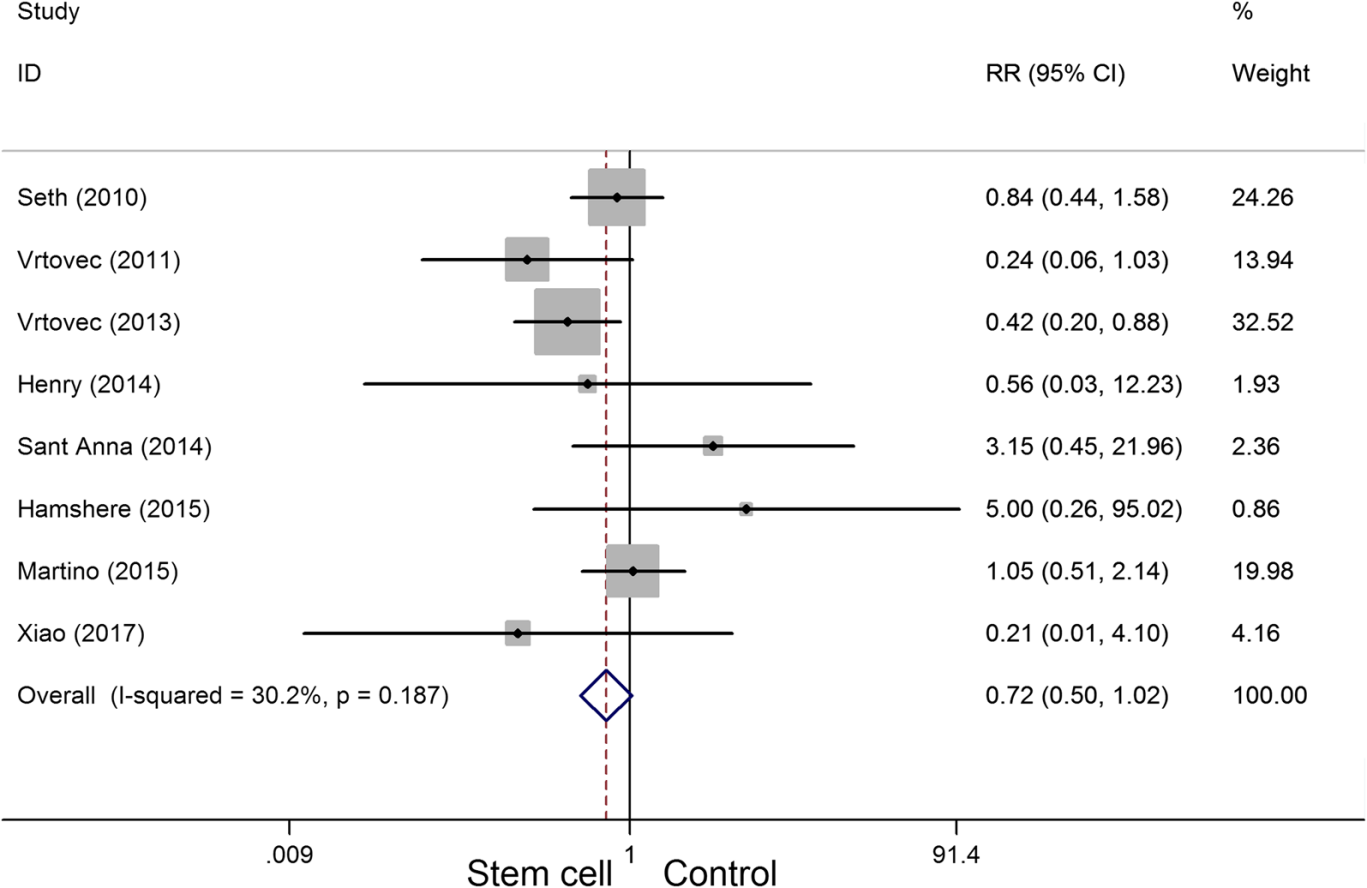
Forest plot of the mortality of stem cell therapy versus controls in patients with dilated cardiomyopathy

Eight articles involving 471 participants presented the mortality data. The heterogeneity test indicated that there was no statistical heterogeneity (*P*_heterogeneity_ = 0.187, *I*^2^ = 30.2%), and there was no significant differences in mortality (RR = 0.72, 95% CI 0.50 to 1.02) (Fig. 4) between the stem cell therapy group and control group.

#### LVEF

**Fig. 5:**
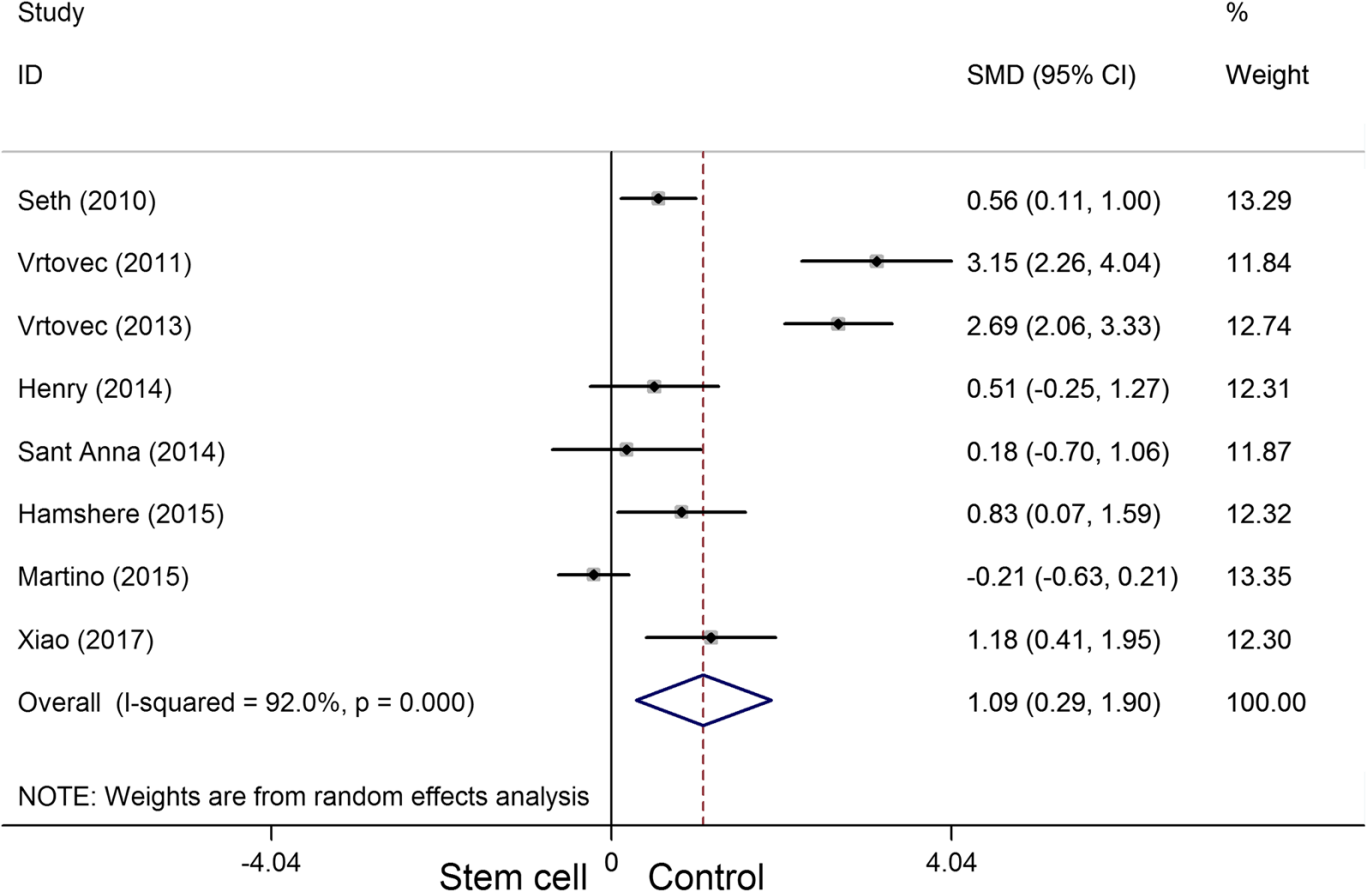
Forest plot of the LVEF of stem cell therapy versus controls in patients with dilated cardiomyopathy

Eight articles involving 398 participants presented the LVEF data. The heterogeneity test indicated that there was significant statistical heterogeneity (*P*_heterogeneity_ < 0.001, *I*^2^ = 92%), and a significant increase in LVEF (SMD = 1.09, 95% CI 0.29 to 1.90) (Fig. 5) was observed in the stem cell therapy group compared with the control group.

#### LVESV

**Fig. 6:**
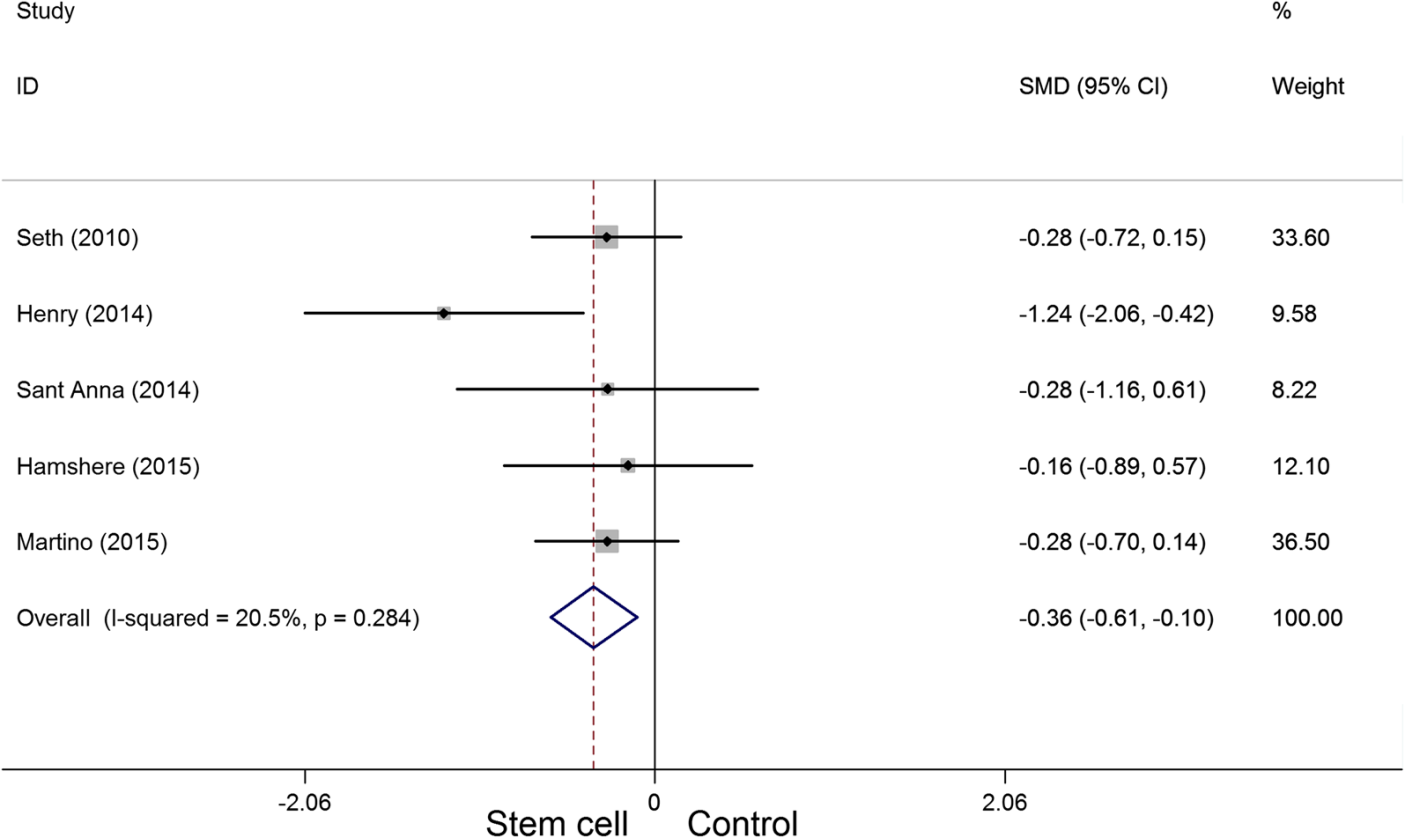
Forest plot of the LVESV of stem cell therapy versus controls in patients with dilated cardiomyopathy

Five articles involving 248 participants presented the LVESV data. The heterogeneity test indicated that there was no statistical heterogeneity (*P*_heterogeneity_ = 0.284, *I*^2^ = 20.5%), and a significant decrease in LVESV (SMD = − 0.36, 95% CI − 0.61 to − 0.10) (Fig. 6) was observed in the stem cell therapy group compared with the control group.

#### LVEDCS

**Fig. 7:**
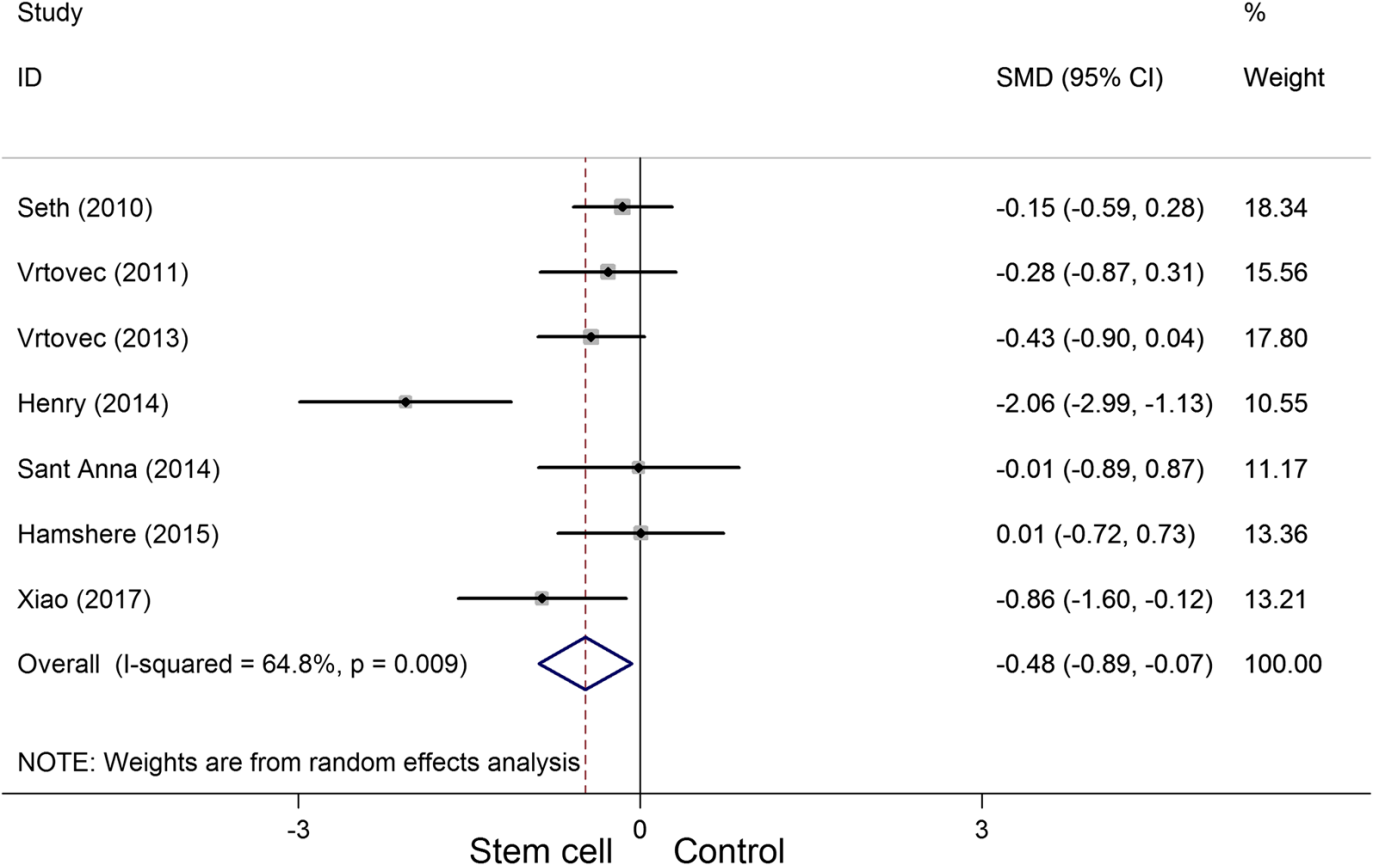
Forest plot of the LVEDCS of stem cell therapy versus controls in patients with dilated cardiomyopathy

Seven articles involving 310 participants presented the LVEDCS data. The heterogeneity test indicated that there was a significant statistical heterogeneity (*P*_heterogeneity_ = 0.009, *I*^2^ = 64.8%), and the outcome showed that a significant decrease in LVEDCS was observed between the two groups (SMD = − 0.48, 95% CI − 0.89 to − 0.07) (Fig. 7).

#### 6-min-walk test

**Fig. 8:**
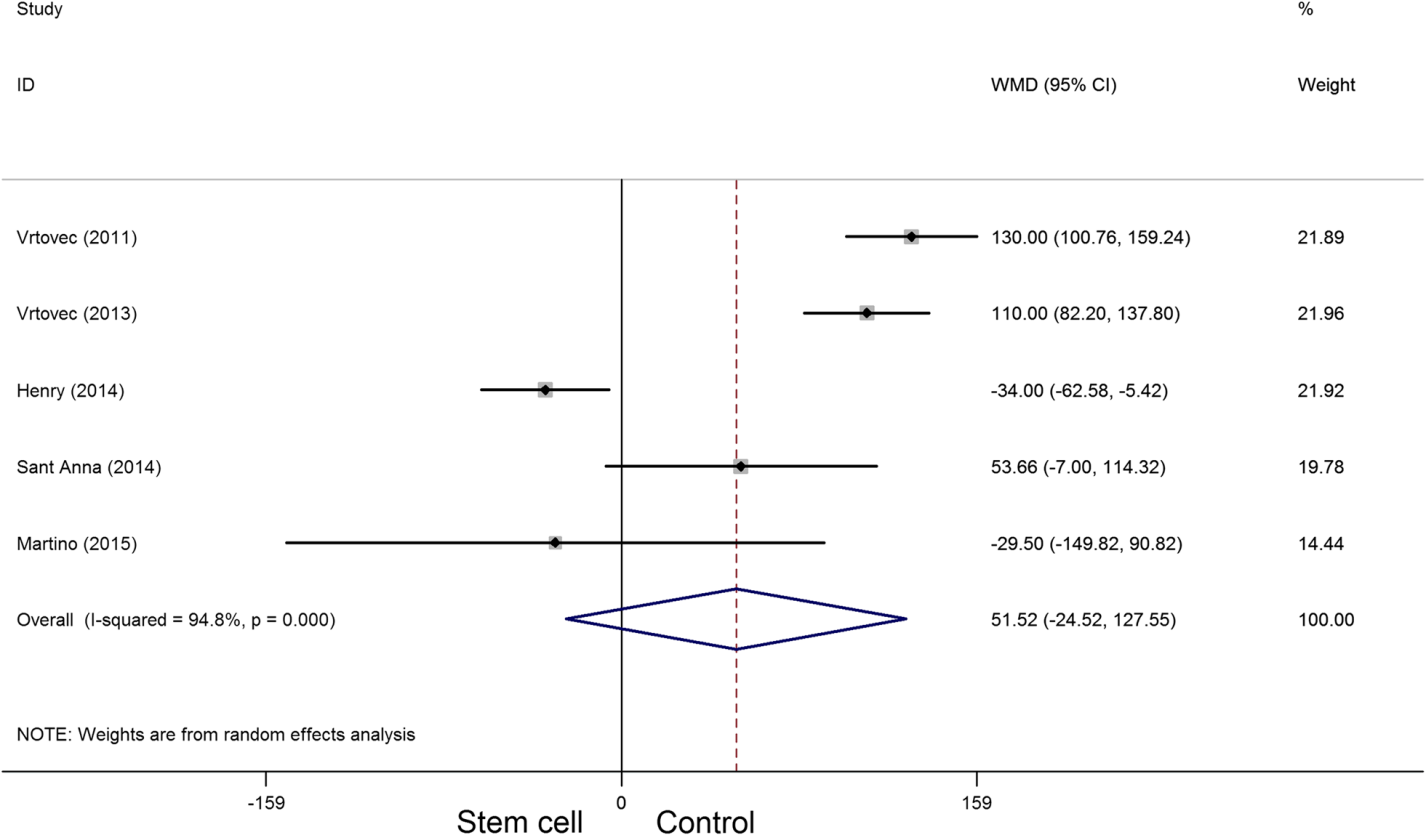
Forest plot of the 6-min-walk test of stem cell therapy versus controls in patients with dilated cardiomyopathy

Five articles involving 384 participants presented the 6-min-walk test data. The heterogeneity test indicated that there was significant statistical heterogeneity (*P*_heterogeneity_ < 0.001, *I*^2^ = 94.8%), and no significant difference was observed in the 6-min-walk test between the two groups (WMD = 51.52, 95% CI − 24.52 to 127.55) (Fig. 8).

### Sensitivity analysis

**Fig. 9:**
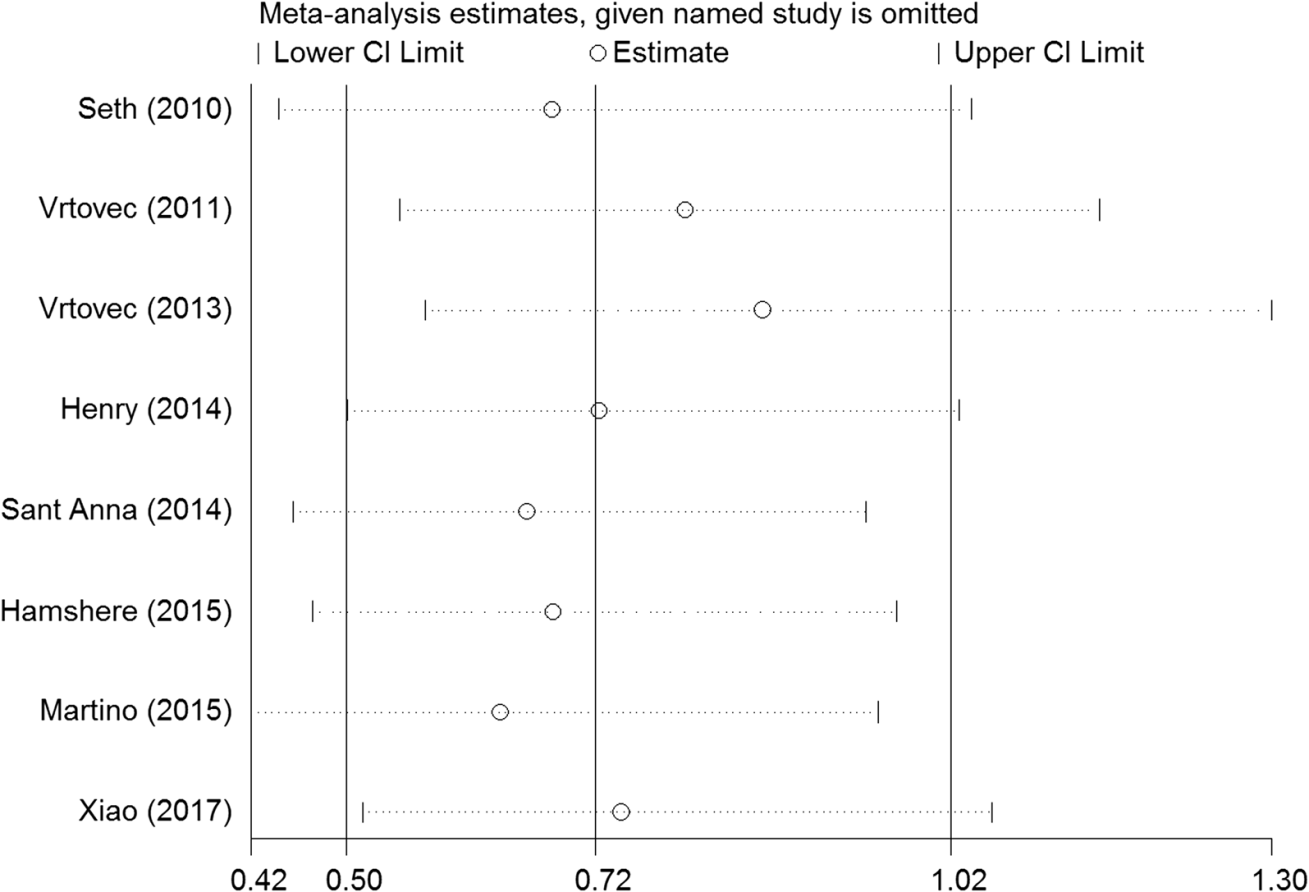
Sensitivity analysis of the mortality of stem cell therapy versus controls in patients with dilated cardiomyopathy

**Fig. 10:**
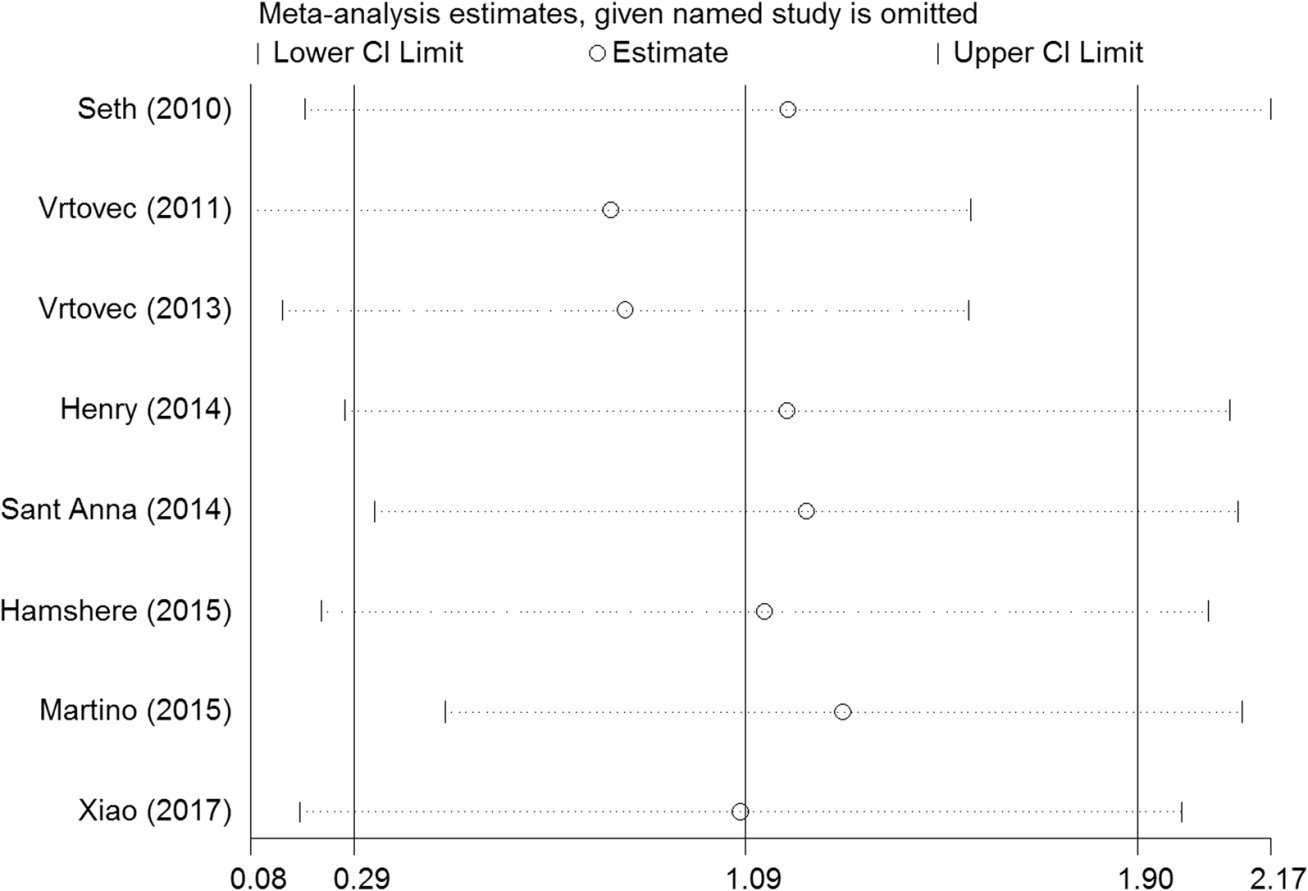
Sensitivity analysis of the LVEF of stem cell therapy versus controls in patients with dilated cardiomyopathy

**Fig. 11:**
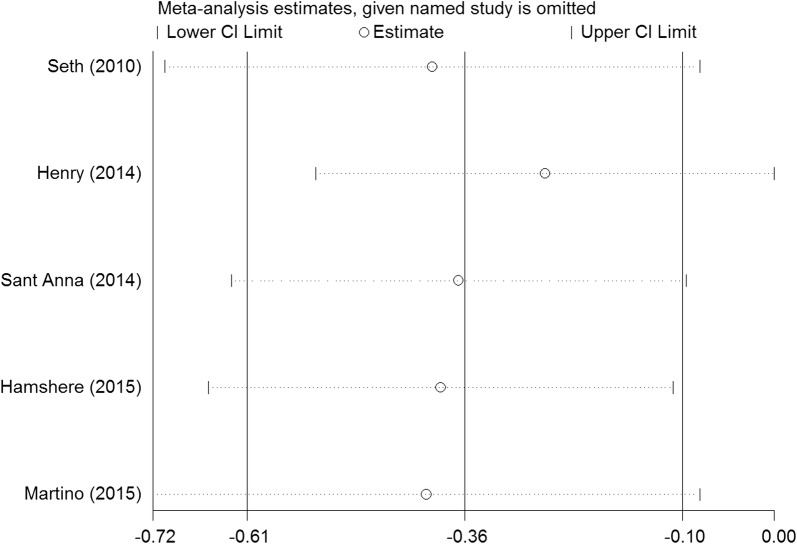
Sensitivity analysis of the LVESV of stem cell therapy versus controls in patients with dilated cardiomyopathy

**Fig. 12:**
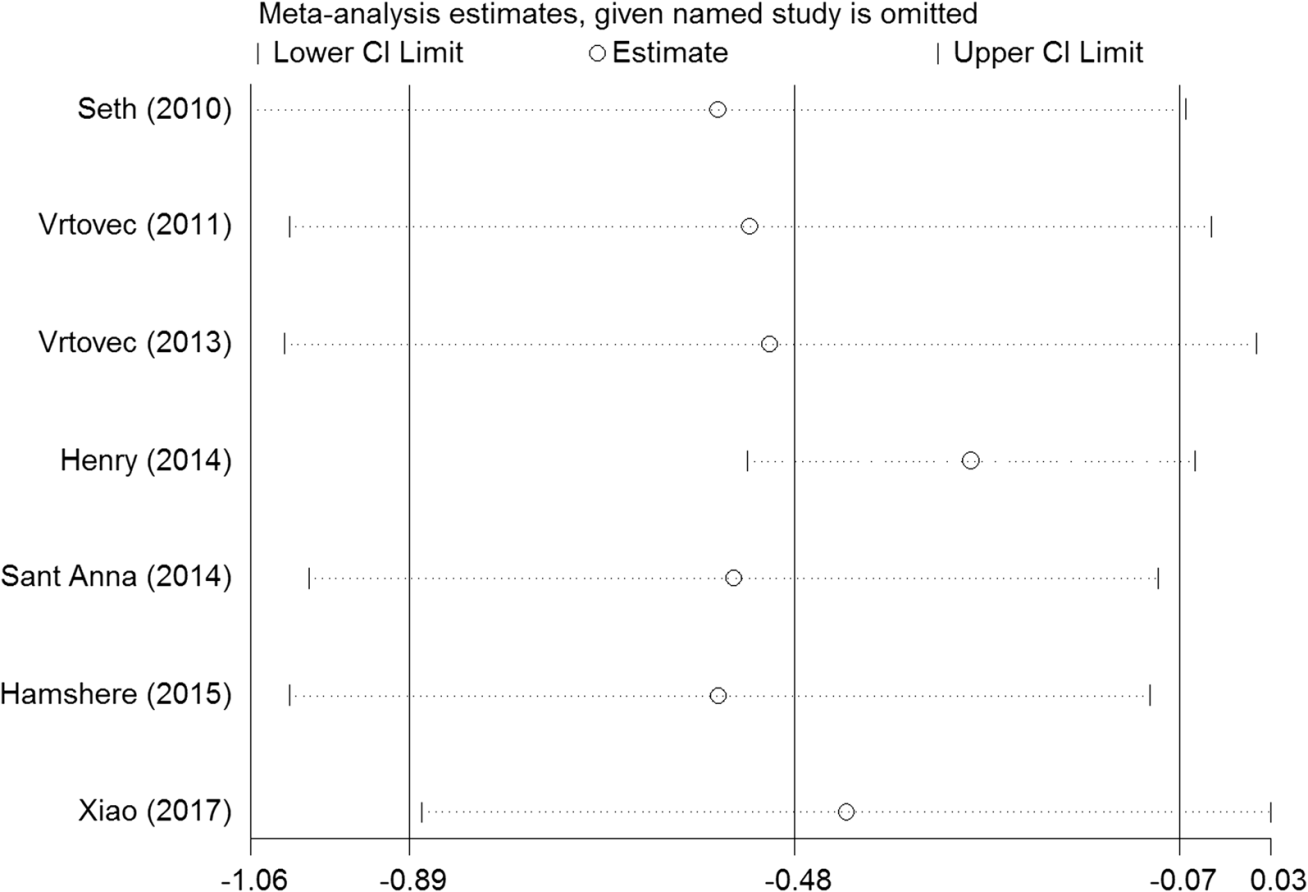
Sensitivity analysis of the LVEDCS of stem cell therapy versus controls in patients with dilated cardiomyopathy

**Fig. 13:**
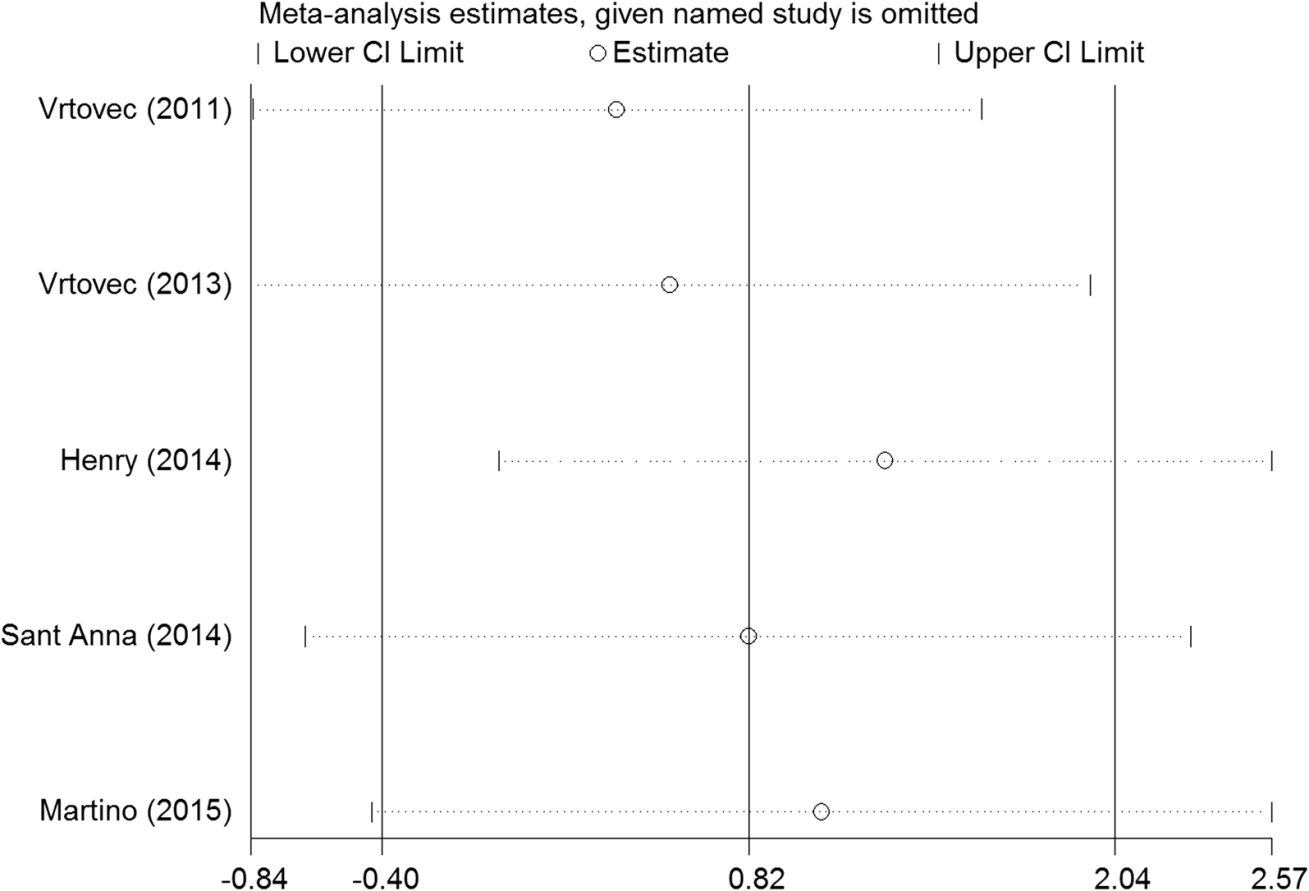
Sensitivity analysis of the 6-min-walk test of stem cell therapy versus controls in patients with dilated cardiomyopathy

We performed a series of sensitivity analyses by sequentially deleting each qualifying study to assess the impact of a single dataset on the pooled results. As seen in Figs. 9, 10, 11, 12, and 13, any individual study was omitted, but the overall statistical significance remained unchanged, suggesting that our results were statistically robust.

### Publication bias

**Fig. 14:**
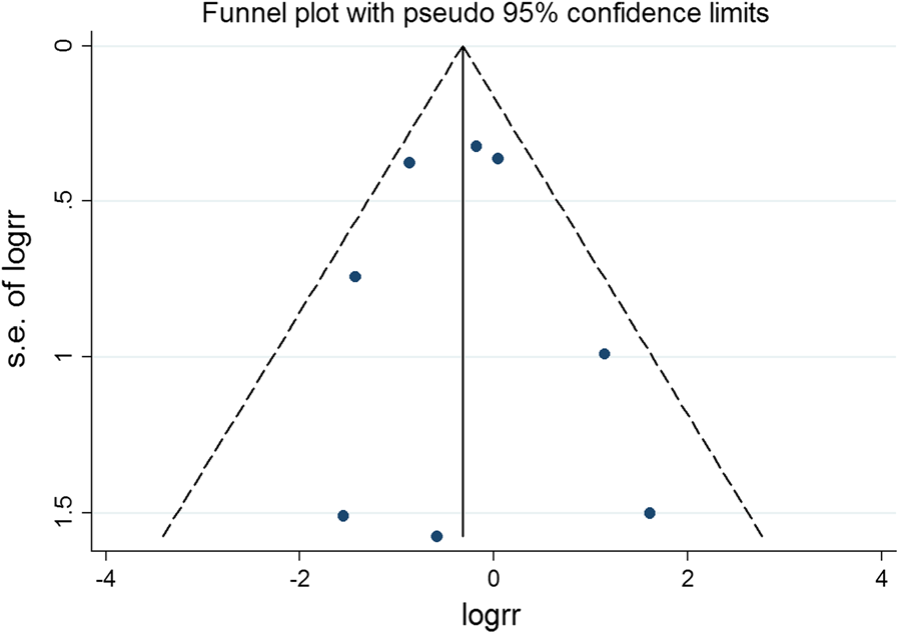
Funnel plot for publication bias test for the mortality of stem cell therapy

**Fig. 15:**
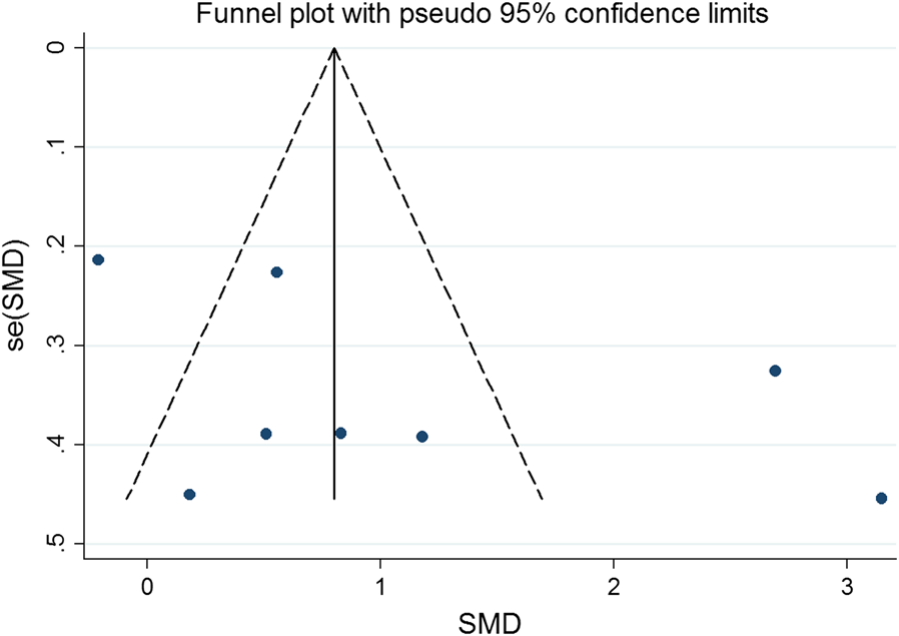
Funnel plot for publication bias test for the LVEF of stem cell therapy

**Fig. 16:**
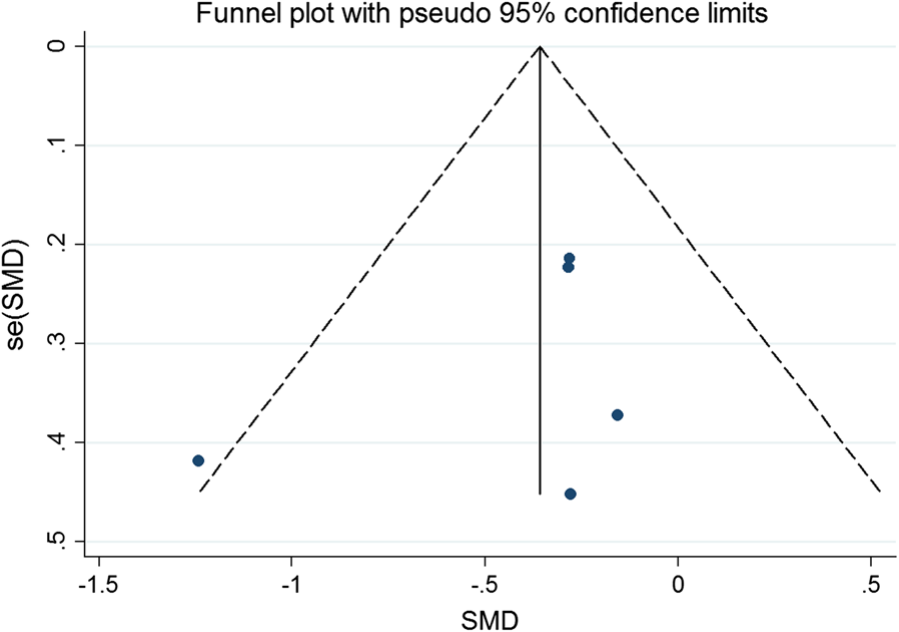
Funnel plot for publication bias test for the LVESV of stem cell therapy

**Fig. 17:**
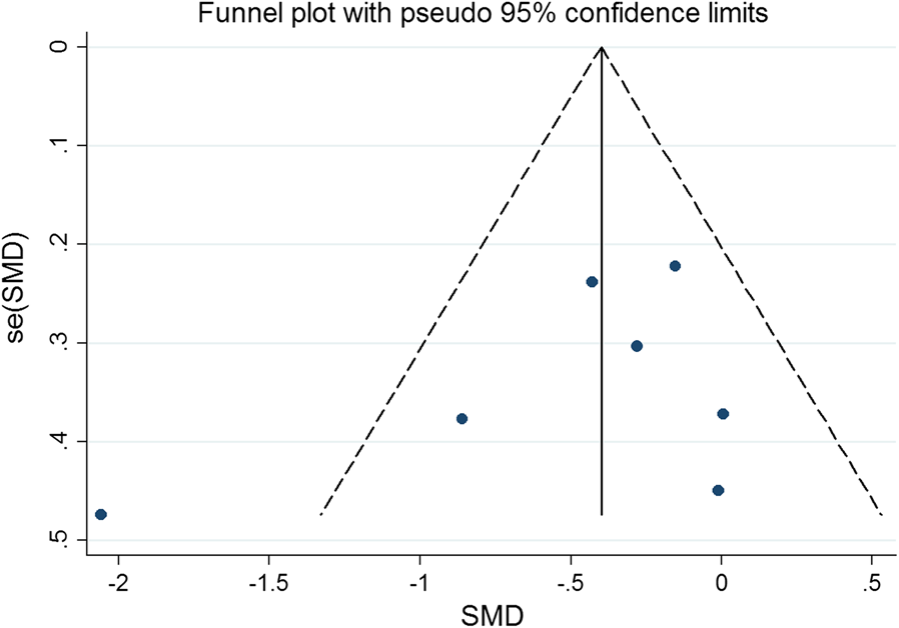
Funnel plot for publication bias test for the LVEDCS of stem cell therapy

**Fig. 18:**
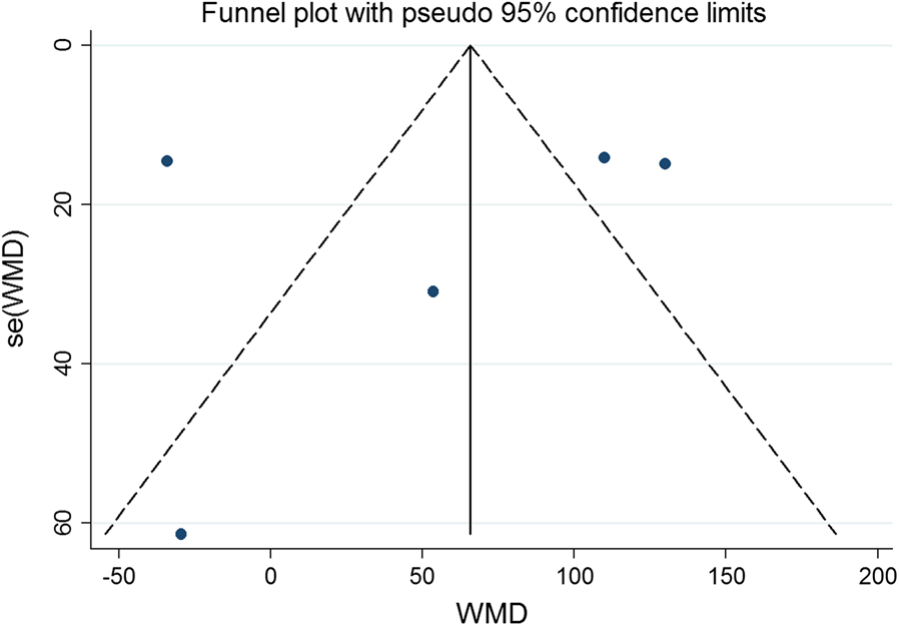
Funnel plot for publication bias test for the 6-min-walk test of stem cell therapy

Publication bias in literature was assessed by funnel plot, Begg’s and Egger’s test. As shown in Figs. 14, 15, 16, 17, and 18, there was no evidence of publication bias for mortality (Begg’s test: *P* = 0.902; Egger’s test: *P* = 0.875), LVEF (Begg’s test: *P* = 0.386; Egger’s test: *P* = 0.425), LVESV (Begg’s test: *P* = 0.462; Egger’s test: *P* = 0.448), LVEDCS (Begg’s test: *P* = 0.230; Egger’s test: *P* = 0.589), and 6-min walk test (Begg’s test: *P* = 0.806; Egger’s test: *P* = 0.417).

## Discussion

In this study, we evaluated the efficacy and safety of stem cell therapy in patients with dilated cardiomyopathy by a systematic appraisal and meta-analysis. Our meta-analysis showed that stem cell therapy improves left ventricular ejection fraction and reduces left ventricular end-systolic volume and left ventricular end-diastolic chamber size in patients with dilated cardiomyopathy. However, stem cell therapy has no effect on mortality and exercise capacity. A promising result has been achieved in this systematic appraisal and meta-analysis; stem cell therapy significantly improved LVEF in terms of left ventricular systolic function. Consistently, the final volume of left ventricular contraction and the decrease in ventricular diameter at the end of left ventricular diastolic were observed. Recent studies [27, 28] have also shown that stem cell transplantation can prevent remodeling and stimulate reverse remodeling of left ventricular.

The efficacy and safety of stem cell therapy in patients with dilated cardiomyopathy have been investigated by previous meta-analysis. Recently, Jiao et al. [29] conducted a systematic review and meta-analysis about the efficacy of stem cell therapy in dilated cardiomyopathy. Jiao’s study found that stem cell therapy has no effect on LVEDCS; however, our study showed that stem cell therapy significantly improved LVEF and reduced LVESV and LVEDCS. Between-trial heterogeneity is a common problem when interpreting the results of meta-analyses. Heterogeneity was found in the overall comparisons in this study; hence, the random-effects model was used. Different patient selection criteria, intervention options, and time intervals may have influenced the interpretation of heterogeneity.

Dilated cardiomyopathy is an important cause of heart failure. It is a primary myocardial disease with unknown pathogenesis, accompanied by a large number of cardiomyocytes loss, and fibroblast replacement, known as ventricle remodeling [1, 30]. Therefore, the recovery of non-functional cardiomyocytes is further studied and gained much attention. Beltrami et al. [31] found that myocardial cells were not terminal differentiated cells; however, the number of regenerative cells was far less than the number needed for cardiac repair. Exogenous functional cells transplantation replaces, repairs, or enhances the biological function of non-functional cardiomyocytes, i.e., cell development and exploration based on myocardial regeneration therapy, is a promising new strategy for the treatment of cardiovascular diseases [32, 33]. Although global LVEF has been used as the gold standard for measuring cardiac function, especially in large trials such as the CADILLAC trial [34, 35], its use in cell therapy trials remains controversial [36]. LVEF is an important predictor of mortality in patients with left ventricular dysfunction [37], the cohort of patients included in BMSC trials after AMI [38, 39]. Consistent with previous studies, we observed a certain degree of improvement in LVEF in this systematic review and meta-analysis, which is conducive to stem cell therapy. Our results demonstrated that stem cell therapy improved LVEF and LVESV in patients with dilated cardiomyopathy. However, stem cell therapy has no effect on mortality (RR = 0.72, 95% CI 0.50 to 1.02) and 6-min-walk test (WMD = 51.52, 95% CI − 24.52 to 127.55). According to the effect value of the RR and WMD, there was a change trend in favor of stem cell therapy group, although neither of them reached statistical significance. We speculate that this may be related to the small sample size of the included trials (the total sample is less than 300 cases in stem cell therapy group).

At the same time, we noted some limitations in this meta-analysis. Firstly, meta-analysis may be biased when the literature search does not identify all relevant trials or subjectively apply the selection criteria for the trial. To minimize these risks, we conducted thorough searches in multiple bibliographic databases and used clear criteria for research selection, data extraction, and data analysis. Secondly, language can also lead to bias. Specifically, we only chose articles published in English so that other qualified studies published in other language may be excluded. Finally, only a few qualified studies were included. Finally, the number of studies included was relatively small. Only four studies had more than 50 participants in the experimental group in the included studies. The number of participants in most studies was small, so there was a risk of small research biases that could lead to exaggerated effects.

Considering the above problems and limitations, more rigorous clinical randomized controlled trials with larger samples are needed to further verify the role of stem cell therapy in dilated cardiomyopathy patients in the future.

## Conclusions

This meta-analysis suggests that stem cell therapy improves LVEF and reduces LVESV and LVEDCS in patients with dilated cardiomyopathy. However, future well-designed large studies are warranted to clarify the effect of stem cell therapy in patients with dilated cardiomyopathy.

## Data Availability

Not applicable.

## Abbreviations

WMD: weighted mean difference
SMD: standard mean difference
RR: relative risk
CI: confidence interval
LVEF: left ventricular ejection fraction
LVESV: left ventricular end-systolic volume
LVEDCS: left ventricular end-diastolic chamber size
RCT: randomized controlled trial
